# Evaluation of Insulin-like Growth Factor-1 and Insulin-like Growth Factor Binding Protein-3 Expression Levels in Patients with Chronic Lymphocytic Leukemia

**DOI:** 10.4274/tjh.2016.0075

**Published:** 2016-12-01

**Authors:** Mesut Ayer, Abdullah Sakin, Selim Ay, Aylin Ayer, Elif Gökçen Sazak, Melih Aktan

**Affiliations:** 1 Haseki Training and Research Hospital, Clinic of Hematology, İstanbul, Turkey; 2 Okmeydanı Training and Research Hospital, Clinic of Internal Medicine, Oncology Unit, İstanbul, Turkey; 3 Haseki Training and Research Hospital, Clinic of Internal Medicine, İstanbul, Turkey; 4 İstanbul University İstanbul Faculty of Medicine, Department of Internal Medicine, Division of Hematology, İstanbul, Turkey

**Keywords:** Chronic lymphocytic leukemia, Insulin-like growth factor-1, Insulin-like growth factor binding protein-3

## Abstract

**Objective:**

Chronic lymphocytic leukemia (CLL) is a disease of nonproliferating and mature-appearing B lymphocytes. Insulin-like growth factor-1 (IGF-1) is a small peptide hormone and has mitogenic and antiapoptotic effects, and insulin-like growth factor binding protein-3 (IGFBP-3) has antiproliferative effects on cells. In this study, we investigated plasma levels of both IGF-1 and IGFBP-3 in patients with CLL compared with controls, and we compared these plasma levels according to prognostic factors.

**Materials and Methods:**

Patients with newly diagnosed CLL who were being followed at the Haseki Training and Research Hospital, İstanbul, Turkey, and volunteers were included in this study. Patients were stratified according to the Rai staging system. Statistical analysis was conducted using SPSS 17.0 for Windows.

**Results:**

Forty-three patients [16 women (37%) and 27 men (63%)] were enrolled in this study. Twenty-one volunteers (11 women, 10 men) were included in the control group. The median age of the patients was 65±9 years (range: 18-63 years), and subjects in the control group were 68±8 years old (range: 18-63 years). Even though the plasma levels of IGF-1 were higher and those of IGFBP-3 were lower and the ratio of IGF-I/IGFBP-3 was higher in comparison with the control group, these differences were not statistically significant (p>0.05). In the study group, IGF-1 levels appeared to be increased in parallel to more advanced Rai stages. There were no significant differences between the other groups (p=0.105).

**Conclusion:**

Plasma IGF-I levels were found higher in patients than in the control group and plasma IGFBP-3 levels were lower; however, neither result was statistically significant. Plasma IGF level increment was observed in concordance with Rai staging. These results prompted us to think that plasma IGF-1 levels in CLL patients are correlated with tumor burden and Rai staging and therefore could be a valuable prognostic factor. Further comprehensive studies are required to support our results.

## Abstract (tr)

**Amaç:**

Kronik lenfositik lösemi (KLL) olgun görünümlü B lenfositlerin hastalığıdır. İnsülin-benzeri büyüme faktörü-1 (IGF-1), mitojenik ve antiapoptotik etkili küçük peptid hormondur ve insülin-benzeri büyüme faktörü bağlayıcı protein-3 (IGFBP-3) ise hücre üzerinde antiproliferative etki gösterir. Çalışmamızda, KLL hasta grubu ve kontrol grubunda plazma IGF-1 ve IGFBP-3 düzeylerini ve prognostik faktörlerle ilişkisini karşılaştırdık.

**Gereç ve Yöntemler:**

Haseki Eğitim ve Araştırma Hastanesi, Hematoloji Bölümü’nde takip edilen yeni tanı almış KLL hastaları ile kontrol grubu çalışmaya dahil edilmiştir. Hastalar Rai sistemine göre evrelendirilmiştir. İstatistiksel analiz SPSS for Windows version 17.0 kullanılarak yapılmıştır.

**Bulgular:**

Kırk üç hasta [16 kadın (%37) ve 27 erkek (%63)] çalışmaya alınmıştır. Kontrol grubu 21 kişiden (11 kadın, 10 erkek) oluşmuştur. Hasta grubunda ortanca yaş 65±9 (18-63), kontrol grubunda 68±8’dir (18-63). Kontrol grubu ile karşılaştırıldığında; çalışma grubunda plazma IGF-1 düzeyi yüksek; IGFBP-3 düzeyi düşük, IGF-I/IGFBP-3 oranı ise yüksek olarak bulunmasına rağmen istatistiksel yönden anlamlı değildi (p>0,05). Çalışma grubunda plazma IGF-1 düzeyi yüksekliği ile Rai ileri evresi paralellik gösteriyordu. Diğer gruplarla istatistiksel yönden anlamlı farklılık yoktu (p=0,105).

**Sonuç:**

Çalışma grubunda, plazma IGF-1 düzeyi kontrol grubundan daha yüksek, IGFBP-3 düzeyi ise düşük bulundu, bununla beraber istatistiksel yönden anlamlı farklılık yoktu. Plazma IGF-1 düzeyi yüksekliği ile Rai ileri evresi uyumlu idi. Bu sonuçlar bize, IGF-1 düzeyinin KLL hastalarında tümor yükü ve Rai evresi ile ilişkili olduğunu ve prognostik faktör olarak değerli olabileceğini düşündürmüştür. Bu sonuçları destekleyebilmek için daha geniş kapsamlı çalışmalara ihtiyaç vardır.

## INTRODUCTION

Chronic lymphocytic leukemia (CLL) is a disease of nonproliferating and mature-appearing B lymphocytes. Most patients with CLL are elderly; just 10% are aged less than 50 years. In the evaluation of the prognosis of patients with CLL, mutations and cytogenetic abnormalities are crucial and independent markers in addition to clinical classifications [1,2].

Insulin-like growth factor (IGF) has a pivotal role in the normal development of fetuses and children. In adulthood, this growth factor has a role in the inhibition of cell proliferation and apoptosis, in addition to its role in cellular metabolism. IGF-1 is a small peptide hormone that has mitogenic and antiapoptotic effects, but IGF-binding protein 3 (IGFBP-3) has an antiproliferative effect and negates the mitogenic effects of IGF-1 by stimulating apoptosis. In several types of tumors, it has been shown that IGF-1 levels are increased and IGFBP-3 levels are decreased [3,4].

In this study, we investigated plasma levels of both IGF-1 and IGFBP-3 in patients with CLL compared with controls and we compared these plasma levels according to prognostic factors.

## MATERIALS AND METHODS

Patients who were newly diagnosed with CLL at the Haseki Training and Research Hospital and healthy volunteers were included in this study. Patients with the following conditions were excluded from the study: chronic renal disease, decompensated heart failure, chronic hepatitis, coronary artery disease, diabetes mellitus with organ damage or uncontrolled plasma glucose levels, chronic inflammatory disease, and major trauma in the last the year. Twenty-one volunteers were included in the control group.

Plasma samples were obtained after centrifugation at 3500 rpm for 8 min and stored at -80 °C until analysis. Plasma IGF-I detection was performed using an ELISA kit (DRG International, USA) in accordance with the manufacturer’s protocol. The sensitivity of the kit was 0.15 ng/mL. IGFBP-3 levels were measured using a BioSource ELISA Kit with solid-phase enzyme immunoassay (BioSource, Belgium). The analytical sensitivity of the kit was 10 μg/mL. Samples were measured using an ELISA microplate reader (DV-990 BV4, N.T. Laboratory, Italy).

Statistical analysis was conducted using SPSS 17.0 for Windows (SPSS Inc., Chicago, IL, USA). The Kolmogorov-Smirnov test was used to determine whether the samples were from a population with normal distribution. In the comparison of values between two groups, Student’s t-test was used if the group was distributed normally. If the group was not normally distributed, the Mann-Whitney U test was used. In the analysis of proportional data, the chi-square test was used. Pearson and Spearman correlation tests were used to compare numerical parameters. One-way ANOVA testing was used to compare more than two groups; post hoc Bonferroni testing was used for multiple comparisons. In all statistical assessments, the cut-off level of statistical significance was assumed as p<0.05.

Study assessment and methods were approved by the local institutional ethics committee. Patient demographics and laboratory data were obtained from patient records upon obtaining oral informed consent from the patients and their information was recorded.

## RESULTS

Forty-three patients [16 women (37%) and 27 men (63%)] were enrolled in the study. Twenty-one control subjects who were demographically compatible (11 women, 10 men) were included in the control group. The median age of the patient and control groups was 65±9 years (range: 18-63 years) and 68±8 years (range: 18-63 years), respectively. There was no statistical demographic difference between the two groups (p>0.05).

Among the patients, 44% (n=19) had CLL of Rai stage 0, 11% (n=5) stage I, 20% (n=9) stage II, 16% (n=7) stage III, and 6% (n=3) stage IV.

Compared with the control group, IGF-1 levels were found to be higher in the study group (531±246 ng/mL), and IGF-1 levels were also detected to be subsequently higher in every Rai stage (Rai stage 0, 1… etc.). However, this difference was not statistically significant (p>0.05). IGFBP-3 levels were found lower in the study group (3890±324 ng/mL) and IGFBP-3 levels were also found lower in each sequential Rai stage (Rai stage 0, 1… etc.), but this was not statistically significant (p>0.05). The IGF-I/IGFBP-3 ratio was higher in the study group (0.32±0.60 ng/mL), although the difference was not statistically significant (p=0.5) (Table 1).

**Figure 1:**
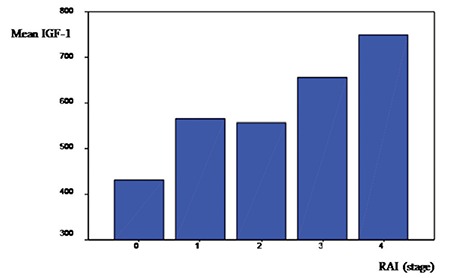
Mean insulin-like growth factor-1 values in Rai stages.

**Figure 2:**
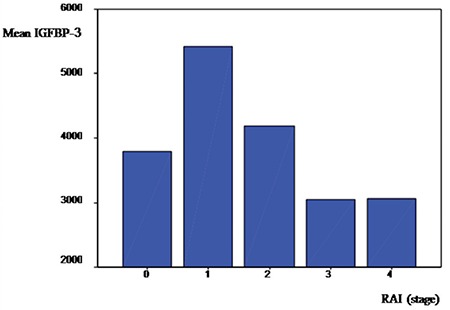
Insulin-like growth factor binding protein-3 values in Rai stages.

In patients with CLL, we observed that IGF-1 levels had a positive correlation with Rai stages (Rs=0.411; p<0.01). The same correlation was not observed for IGFBP-3 levels (Rs=-0.075; p=0.6). IGF-1 levels in the patients with CLL appeared to increase in parallel with more advanced Rai stages. There were no significant differences among Rai stages (p=0.105). When IGFBP-3 levels were compared according to stage, there were also no statistical significant differences (p=0.3) (Figures 1 and 2).

## DISCUSSION

The IGF system plays a pivotal role in normal growth throughout fetal and childhood development. In adult life, this system continues to function by regulating normal cellular metabolism, proliferation, and differentiation and it protects against apoptotic signals. However, aberrant stimulation can contribute to the development and progression of malignant growth [4,5,6].

It has been shown in cell cultures that IGFBP-3 inhibits DNA synthesis without IGF. It has been claimed that IGFBP-3 may link p53 to potential novel autocrine/paracrine signaling pathways and to processes regulated by or dependent on IGF(s), such as cellular growth, transformation, and survival. It has also been asserted that induction of IGFBP-3 gene expression by wild-type, but not mutant, p53 was associated with enhanced secretion of an active form of IGFBP-3 capable of inhibiting mitogenic signaling by IGF-1 [7].

Many studies have reported that high levels of IGF-I, low levels of IGFBP-3, or increments of the molar ratio of IGF-I/IGFBP-3 were associated with various types of cancers [8,9,10,11].

In our study, the mean plasma IGF-I levels of the patients were found higher than those of the control group, but the difference was not statistically significant. Plasma IGFBP-3 level was lower than in the control group, but this was also not statistically significant. However, plasma IGF level increments were in parallel with Rai stages. A reverse correlation was not observed for IGFBP-3 levels.

Molica et al. measured serum levels of IGF-1 and IGFBP-3 in 77 patients with CLL and found them to be statistically significantly lower than in the control group. However, no significant correlation was found between serum levels of either IGF-1 or IGFBP-3 and clinicohematologic variables including age, sex, Rai clinical stages, serum levels of lactate dehydrogenase and beta-2 microglobulin, peripheral blood lymphocyte count, and lymphocyte doubling time [11].

In our study IGF-1 and IGFBP-3 levels were lower in patients with Rai stage 0 compared with the control group. Although IGF-1 levels were found lower in early stages, they increased significantly in parallel with more advanced Rai stages.

In conclusion, plasma IGF-I levels in CLL patients were found higher than in the control group and plasma IGFBP-3 levels were lower. However, neither result was statistically significant. The increments of plasma IGF-1 level were in parallel with Rai staging.

These results suggest that plasma IGF-1 levels in patients with CLL are correlated with tumor burden and Rai staging and therefore might be a valuable prognostic factor. Further comprehensive studies are required to support our results.

## Ethics

Ethics Committee Approval: Study assessment and methods were approved by the local institutional ethics committee (21.01.2009/11); Informed Consent: Patient demographics and laboratory data were obtained from patient records upon obtaining oral informed consent from the patients and their information was recorded.

## Figures and Tables

**Table 1:**
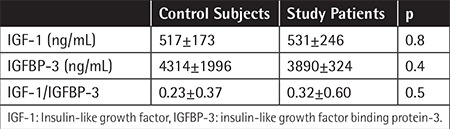
Insulin-like growth factor-1 and insulin-like growth factor binding protein-3 values.
